# Fast Healthcare Interoperability Resources (FHIR) as a Meta Model to Integrate Common Data Models: Development of a Tool and Quantitative Validation Study

**DOI:** 10.2196/15199

**Published:** 2019-10-16

**Authors:** Emily Rose Pfaff, James Champion, Robert Louis Bradford, Marshall Clark, Hao Xu, Karamarie Fecho, Ashok Krishnamurthy, Steven Cox, Christopher G Chute, Casey Overby Taylor, Stan Ahalt

**Affiliations:** 1 North Carolina Translational and Clinical Sciences Institute University of North Carolina at Chapel Hill Chapel Hill, NC United States; 2 Renaissance Computing Institute University of North Carolina at Chapel Hill Chapel Hill, NC United States; 3 Johns Hopkins University Baltimore, MD United States

**Keywords:** health information interoperability, electronic health records, data sharing, controlled vocabularies

## Abstract

**Background:**

In a multisite clinical research collaboration, institutions may or may not use the same common data model (CDM) to store clinical data. To overcome this challenge, we proposed to use Health Level 7’s Fast Healthcare Interoperability Resources (FHIR) as a meta-CDM—a single standard to represent clinical data.

**Objective:**

In this study, we aimed to create an open-source application termed the Clinical Asset Mapping Program for FHIR (CAMP FHIR) to efficiently transform clinical data to FHIR for supporting source-agnostic CDM-to-FHIR mapping.

**Methods:**

Mapping with CAMP FHIR involves (1) mapping each source variable to its corresponding FHIR element and (2) mapping each item in the source data’s value sets to the corresponding FHIR value set item for variables with strict value sets. To date, CAMP FHIR has been used to transform 108 variables from the Informatics for Integrating Biology & the Bedside (i2b2) and Patient-Centered Outcomes Research Network data models to fields across 7 FHIR resources. It is designed to allow input from any source data model and will support additional FHIR resources in the future.

**Results:**

We have used CAMP FHIR to transform data on approximately 23,000 patients with asthma from our institution’s i2b2 database. Data quality and integrity were validated against the origin point of the data, our enterprise clinical data warehouse.

**Conclusions:**

We believe that CAMP FHIR can serve as an alternative to implementing new CDMs on a project-by-project basis. Moreover, the use of FHIR as a CDM could support rare data sharing opportunities, such as collaborations between academic medical centers and community hospitals. We anticipate adoption and use of CAMP FHIR to foster sharing of clinical data across institutions for downstream applications in translational research.

## Introduction

### Background

The proliferation of common data models (CDMs) for electronic health record (EHR) data has had a positive impact on cross-institutional data sharing and large-scale participant recruitment [1-3]. At present, the 3 major clinical CDMs in use by the academic community are Informatics for Integrating Biology & the Bedside (i2b2) [4], Patient-Centered Outcomes Research Network (PCORnet) [5], and Observational Medical Outcomes Partnership (OMOP) [6], each of which uses a slightly different architecture to achieve the same result: to represent and store EHR data in a relational database. The ability to query common data structures and provision data to collaborators in a shared format reduces the burden on data analysts and enforces common definitions that allow clinical data to be appropriately merged and compared across institutions. However, despite these affordances, there is no guarantee that all institutions involved in a multisite collaboration are using the same CDM, potentially negating the advantage.

The challenge of cross-institutional sharing of clinical data has risen in the context of the Biomedical Data Translator program [7-9], funded by the National Center for Advancing Translational Sciences. The Translator program aims “to design and prototype a ‘Translator’ system capable of integrating existing biomedical data sets...and ‘translating’ those data into insights that can accelerate translational research, support clinical care, and leverage clinical expertise to drive research innovations” [8]. Clinical data are central to the program and critical for its success. Yet, despite the importance of clinical data, Translator teams have not adopted a uniform CDM to enable the sharing of clinical data across the consortium. Moreover, even if current Translator teams were to adopt a uniform CDM, (1) future Translator collaborators and users may not be positioned to support the agreed-upon model and (2) the dynamic and complex nature of clinical data could render an agreed-upon model quickly obsolete.

### Common Data Models: Current State

The challenge of cross-institutional EHR data sharing is by no means limited to the Translator program [10]. Institutions wishing to engage in data sharing may not support the same CDM—perhaps one uses i2b2, whereas another uses OMOP. In such cases, it is likely not possible for one institution to simply agree to stand up a new CDM to accommodate the other institution. Mapping institutional EHR data to any one of the major CDMs is resource- and personnel-intensive and requires an ongoing commitment to maintain and refresh infrastructure and data over time. The North Carolina Translational and Clinical Sciences Institute (NC TraCS), home of University of North Carolina at Chapel Hill’s National Institutes of Health-funded Clinical and Translational Science Award (CTSA), participates in 2 i2b2-powered networks (CTSA Accrual to Clinical Trials [ACT] and the Carolinas Collaborative) and one PCORnet-powered network (Stakeholders, Technology, and Research Clinical Research Network [STAR CRN]). In a poll of STAR collaborators, we discovered that maintaining CDM infrastructure consumes, on average, just over 1 full-time equivalent (FTE) per CDM per year. Initial implementation effort varies by CDM, but ranged from 0.8 to 3.8 FTE in our poll (see Table 1). As institutions are asked to adopt more CDMs (and the number of available CDMs multiply), this level of effort increases and can quickly become untenable, even with existing expertise, education, and documentation. Moreover, as can be seen in Table 1, the effort expended can differ greatly between sites, with some sites needing to expend far more resources than others to achieve the same goals.

The core function of a CDM is to enable clinical data harmonization and interoperability. CDMs thus share a goal with Health Level 7’s Fast Healthcare Interoperability Resources (HL7’s FHIR), a health care data representation standard increasingly supported by major EHR vendors. In FHIR, clinical data are split into *resources* or data domains. As of version 4.0.0, FHIR provides 22 nondraft-status Base resources, such as Patient, Practitioner, and Encounter, and 33 nondraft-status Clinical resources, such as Procedure, Observation, and MedicationRequest. Other types of FHIR resources include Foundation, Financial, and Specialized [11]. Each resource comprises structured fields that describe the resource—for example, the Encounter resource contains fields to capture the type of encounter, length of stay, and discharge disposition. In addition to defining specific resources and fields, FHIR enforces the use of established code sets (eg, LOINC, SNOMED CT, and ICD-9/-10) or FHIR-specific value sets in many of its fields to maximize standardization. Where provided fields and code sets are not sufficient, FHIR offers the ability for individual users and organizations to build *extensions* to the standard to capture data that are not explicitly defined by HL7 [12]. Considering these characteristics, FHIR can also be considered a CDM [13].

A testament to the connection between FHIR and CDMs is the fact that several efforts have already been initiated to map either i2b2, PCORnet, or OMOP to FHIR [14-19]. The software applications described in these previous studies each map a single-source data model to FHIR; to be able to map additional data models, a user must use multiple applications.

**Table 1:** Effort expended by Stakeholders, Technology, and Research Clinical Research Network sites to stand up and maintain common data models.

Site^a^ and number of common data models (CDMs) currently maintained		Number of full-time equivalent (FTE) to stand up *one* new CDM	Number of FTE to maintain all CDMs
**University of North Carolina** **Chapel Hill**			
	3 (PCORnet^b^, i2b2^c^ [2 separate ontologies])	Informatics: 1.0	Informatics: 2.0
		Project Management: 0.5	Project Management: 1.0
**Site 1**			
	3 (PCORnet, i2b2, OMOP^d^)	Informatics : 2.3	Informatics: 5.0
		Project Management: 1.5	Project Management: 2.0
**Site 2**			
	3 (PCORnet, i2b2 [2 separate ontologies])	Total: 2.5	Informatics: 3.0
			Project Management: 2.0
**Site 3**			
	6 (PCORnet, i2b2, OMOP, 3 regional models)	Informatics: 2.5	Informatics: 3.0
		Project Management: 0.3	Project Management: 0.3
**Site 4**			
	2 (PCORnet, i2b2)	Total: 0.8	Total: 0.6

^a^Non-University of North Carolina STAR sites have been masked. Sites that did not differentiate between project management and informatics FTE have their effort reported as “Total.”

^b^Patient-Centered Outcomes Research Network.

^c^Informatics for Integrating Biology & the Bedside.

^d^Observational Medical Outcomes Partnership.

### Objective of This Study

Rather than continuing to treat each of these source data models individually, we proposed to improve interoperability, standardization, and semantic harmonization by enabling transformation from any of these (or other) models to FHIR with a single, source-agnostic tool, Clinical Asset Mapping Program for FHIR (CAMP FHIR), that can read from any CDM and map to its straightforward views. This approach will facilitate a multi-institutional collaboration by providing an application that harmonizes across CDMs.

Mapping an individual CDM to FHIR is resource-intensive; collaborating institutions may have *mismatched* CDMs (in terms of model, version, or both); and new CDMs will likely continue to emerge. Although not a replacement for CDMs, on a project-by-project basis, FHIR-formatted data generated by CAMP FHIR can enable easier cross-site data harmonization, supplementing the advances that CDMs have already made in this space. Upon widespread adoption, CAMP FHIR and applications such as these could encourage eventual uptake of FHIR as a *meta-CDM*—a single standard to represent clinical data sourced from any data model.

## Methods

### Designing the Transformation Pipeline

Simply proposing FHIR as yet another CDM for institutions to map their EHRs to would add to, not alleviate, the institutional burden illustrated in Table 1. To avoid this burden and associated costs, we designed CAMP FHIR (and its mapping process) to (1) leverage as much existing CDM mapping and curation work as possible and (2) allow our team to share our mapping work with others, giving other institutions an opportunity to use CAMP FHIR with minimal site-specific changes and resource expenditure required. Thus far, CAMP FHIR has been used to transform data from the i2b2 and PCORnet data models, though the application is designed to allow input from any source data model.

To use FHIR as a meta-CDM, it is important to recognize that unlike i2b2, OMOP, and PCORnet, FHIR was designed to be a standard for data *exchange*—not data persistence. Thus, any CDM-to-FHIR mapping effort involves translating a relational CDM to a serialized format. CAMP FHIR was developed expressly to address this challenge and to make the conversion process as simple as possible.

CAMP FHIR is designed to apply the FHIR standard to EHR data from any source system, although we focused our initial development on the CDMs used at our institution, specifically i2b2 and PCORnet. We began with i2b2, which can be considered a CDM when used with a standard ontology. (University of North Carolina; UNC’s local i2b2 uses a custom ontology and is, thus, not strictly a CDM, but we have also created a version of our i2b2 mapping scripts that uses the i2b2 ACT ontology, which is standardized.) We first profiled the overlap and gaps between the i2b2 schema (version 1.7.10) and the corresponding FHIR resources (version 3.0.1) and then began to map individual variables (see *Mapping Details*, below).

As we mapped, we quickly faced the challenge of accounting for inconsistencies between the out-of-the-box i2b2 schema and our institution’s modified local version. The challenge was compounded by our knowledge that other institutions that use i2b2 have their own local modifications to contend with, although this is less of an issue if the use of standard ontologies is enforced. Regardless, the realization that some site-specific flexibility would be necessary led us to develop CAMP FHIR with the assumption that many local data sources have idiosyncrasies (even if they are CDMs). (As an example, laboratory reference ranges can be very difficult to harmonize, with different organizations storing the range either as a single field or 2 fields [low end and high end], and with or without comparators such as *<* and *>*.) Considering this, it should therefore be the responsibility of the local database layer to model views to conform to CAMP FHIR’s specific input format. Putting the mapping responsibility on the database layer (rather than within the application itself) provides more flexibility and portability, giving the application the capability to interface with any clinical relational database schema. To achieve this architecture, we chose to use the object-relational mapping tool, Hibernate, which is an open-source Java (Oracle) persistence framework for mapping a relational database to an object-oriented domain model.

After completing the mappings for i2b2, we then created a separate set of mappings for the PCORnet CDM (version 4.1). PCORnet is much stricter about its data model than i2b2, not allowing for local variation in structure or code sets. For this reason, the PCORnet CAMP FHIR mappings are especially portable and would require few (if any) changes to run at any site using the PCORnet CDM.

We provide the PCORnet and ACT mappings as *starter scripts* in the CAMP FHIR GitHub repository [20] to acclimate new users to the tool. To use CAMP FHIR, users run these scripts (or their own versions) to create views within their source database that conform to our CAMP FHIR standard, populate a code mapping table for any value sets, and point CAMP FHIR at the database.

### Mapping Details

Using CAMP FHIR to map to FHIR from any source data model involves two major tasks: (1) mapping each source variable to its corresponding FHIR element; and (2) for variables with strict value sets (eg, race, smoking status, and discharge disposition), mapping each item in the source model’s value set to the corresponding FHIR value set item. We completed these tasks for both the i2b2 and PCORnet data models.

Our i2b2 and PCORnet data marts contain data for 2.9 million patients, with data spanning from July 2004 to the present. Our i2b2 data mart has values populated for 100% of the variables supported by the ACT ontology [21]. Our PCORnet data mart supports all version 4.1 tables [22] other than OBS_GEN, DISPENSING, and DEATH_CAUSE.

#### Informatics for Integrating Biology & the Bedside (i2b2)

UNC at Chapel Hill’s local i2b2 implementation contains data in the following domains: patient demographics, encounter details, diagnoses, procedures, point-of-care location, patient vital signs, laboratory tests, medications, clinical observations, social history, and insurer. To map i2b2 to FHIR, a group of 3 informaticians experienced with the underlying structure and data definitions within UNC’s local i2b2 (1) took each unique concept in a given domain (eg, *diagnosis date* from the domain Diagnosis), (2) reviewed the FHIR documentation for the corresponding resource for that domain (eg, FHIR’s condition resource), (3) determined which field within that FHIR resource was the best fit for the source concept, and (4) recorded the suggested mapping for inclusion in one of the CAMP FHIR views.

For variables with strict value sets, an additional step was necessary. If the best-fit FHIR field had its own strict value set, then each value set item in the source set was mapped to its nearest equivalent in the FHIR set. All value set mappings were stored in a single table, which was then loaded into the i2b2 database. An excerpt from this mapping table is provided in Table 2.

To adhere to our goal of standardization, we opted not to create custom value sets for use within FHIR, opting instead to use the exact value sets provided in the FHIR specification. The tradeoff for this strict adherence to standardization is potential loss of data or loss of granularity. Not every source value set item had an equivalent in FHIR. For example, when the source value was *Other* or another generic catch-all, there was generally not a match in the FHIR set. At this time, unmappable items in our source value sets are left null in the FHIR version of the data. There were also several instances where the FHIR value set was less granular than the source dataset, resulting in a loss of detail after mapping. An example is discharge disposition, where UNC’s local value set contains 44 choices, and FHIR’s value set contains 11. The current version of the i2b2 value set mapping table (using the ACT ontology) can be found in the CAMP FHIR GitHub repository [20].

All mapping tasks were divided among the 3 informaticians, with each person’s mappings peer-reviewed by the other 2. After the mappings were finalized, the mapping team defined database views for each mapped domain. As the views themselves are completely independent of the i2b2 data model, even though they were designed during our i2b2 mapping exercise, they ultimately became the generic set of *CAMP FHIR views* that are packaged with the tool for use with any data model.

For i2b2, the views served to transform the data from the native star schema (with the majority of the data stored in a central fact table, OBSERVATION_FACT) to a normalized format more easily consumable by CAMP FHIR. The code snippet in Textbox 1 shows the construction of the OBSLABS_2FHIR view from OBSERVATION_FACT.

For views containing variables that needed value set conversions (eg, smoking status descriptors in the Observation [Vitals] view), we joined to the prepopulated mapping table when creating the view and populated the FHIR version of each value set item rather than the *local* option.

Patient-Centered Outcomes Research NetworkPCORnet mapping proceeded in much the same way as i2b2; each table, variable, and value set was mapped to FHIR following the steps outlined above, and a value set transformation table was loaded into the PCORnet database. The current version of the PCORnet value set mapping table can be found in the CAMP FHIR GitHub repository [20]. The code snippet in Textbox 2 shows the construction of the OBSLABS_2FHIR view from the LAB_RESULT_CM table. (Note the join to our custom PCORNET_FHIR_MAPPING table to transform the value sets for RESULT_MODIFIER and ABN_IND.)

**Table 2:** Excerpt from University of North Carolina’s Informatics for Integrating Biology and the Bedside-Fast Healthcare Interoperability Resources mapping table.

TABLE_CD	COLUMN_CD	LOCAL_IN_CD	FHIR_OUT_CD	FHIR_SYSTEM
VISIT_DIMENSION	INOUT_CD	EMERGENCY	EMER	https://hl7.org/fhir/STU3/v3/ActEncounterCode/vs.html
VISIT_DIMENSION	INOUT_CD	INPATIENT	IMP	https://hl7.org/fhir/STU3/v3/ActEncounterCode/vs.html
VISIT_DIMENSION	INOUT_CD	OUTPATIENT	AMB	https://hl7.org/fhir/STU3/v3/ActEncounterCode/vs.html

**Structured Query Language (SQL) to create the Clinical Asset Mapping Program for Fast Healthcare Interoperability Resources view OBSLABS_2FHIR from Informatics for Integrating Biology & the Bedside 's OBSERVATION_FACT textbox.**

select distinct
 ofc.patient_num||'-'||ofc.encounter_num||'-'||ofc.provider_id||'-'||
    to_char(ofc.start_date, 'DD-MON-YYYY')||'-'||ofc.concept_cd||'-'||
    ofc.instance_num as OBS_IDENTIFIER,
 'Patient/'||ofc.patient_num as OBS_SUBJECT_REFERENCE,
 'Encounter/'||ofc.encounter_num as OBS_CONTEXT_REFERENCE,
 'http://hl7.org/fhir/ValueSet/observation-category' as OBS_CATEGORY_SYST,
 'laboratory' as OBS_CATEGORY_CODE,
 'Laboratory' as OBS_CATEGORY_DISPLAY,
 'http://loinc.org' as OBS_CODE_CODING_SYST,
  ofc.concept_cd as OBS_CODE_CODING_CODE,
  cd.NAME_CHAR as OBS_CODE_CODING_DISPLAY,
  ofc.nval_num as OBS_VALUEQUANTITY_VALUE,
  case when ofc.VALTYPE_CD = 'N' and ofc.TVAL_CHAR = 'E' then null
     when ofc.VALTYPE_CD = 'N' and ofc.TVAL_CHAR = 'L' then '<'
     when ofc.VALTYPE_CD = 'N' and ofc.TVAL_CHAR = 'G' then '>'
     when ofc.VALTYPE_CD = 'N' and ofc.TVAL_CHAR = 'LE' then '<='
     when ofc.VALTYPE_CD = 'N' and ofc.TVAL_CHAR = 'GE' then '>='
     else null end as OBS_VALUEQUANTITY_COMPARATOR,
  ofc.units_cd as OBS_VALUEQUANTITY_CODE,
  case when ofc.VALTYPE_CD = 'T' then ofc.TVAL_CHAR
     else null end as OBS_VALUESTRING,
  ofc.START_DATE as OBS_ISSUED,
  null as OBS_EFFECTIVEDATETIME
from
  observation_fact ofc
     left join concept_dimension cd on ofc.concept_cd=cd.concept_cd
     inner join visit_dimension vd ON vd.encounter_num = ofc.encounter_num
where
  ofc.concept_cd like 'LOINC%'

**Structured Query Language (SQL) to create the Clinical Asset Mapping Program Fast Healthcare Interoperability Resources view OBSLABS_2 Fast Healthcare Interoperability Resources from Patient-Centered Outcomes Research Network's LAB_RESULT_CM table.**

select distinct
  labs.LAB_RESULT_CM_ID as OBS_IDENTIFIER,
  'Patient/'||labs.PATID as OBS_SUBJECT_REFERENCE,
  'Encounter/'||labs.ENCOUNTERID as OBS_CONTEXT_REFERENCE,
  'http://hl7.org/fhir/ValueSet/observation-category' as OBS_CATEGORY_SYST,
  'laboratory' as OBS_CATEGORY_CODE,
  'Laboratory' as OBS_CATEGORY_DISPLAY,
  'http://loinc.org' as OBS_CODE_CODING_SYST,
  LAB_LOINC as OBS_CODE_CODING_CODE,
  null as OBS_CODE_CODING_DISPLAY,
  labs.RESULT_NUM as OBS_VALUEQUANTITY_VALUE,
  nvl(tcc1.FHIR_OUT_CD,null) as OBS_VALUEQUANTITY_COMPARATOR,
  case
    when labs.RESULT_UNIT = 'NI' then null
    else labs.RESULT_UNIT
    end as OBS_VALUEQUANTITY_CODE,
  case
    when labs.RESULT_QUAL = 'NI' then null
    else nvl(labs.RESULT_QUAL,labs.RAW_RESULT)
    end as OBS_VALUESTRING,
  labs.RESULT_DATE as OBS_ISSUED,
  nvl(labs.SPECIMEN_DATE,labs.LAB_ORDER_DATE) as OBS_EFFECTIVEDATETIME,
  case
    when labs.NORM_MODIFIER_LOW IN ('EQ,''GE,''GT,''NO') then
    labs.NORM_MODIFIER_LOW||' '||labs.NORM_RANGE_LOW
    else labs.NORM_RANGE_LOW
    end as OBS_REFRANGE_LOW,
  case
    when labs.NORM_MODIFIER_HIGH IN ('EQ,''GE,''GT,''NO') then
    labs.NORM_MODIFIER_HIGH||' '||labs.NORM_RANGE_HIGH
    else labs.NORM_RANGE_HIGH
    end as OBS_REFRANGE_HIGH,
  nvl(tcc2.FHIR_OUT_CD,null) as OBS_INTERPRETATION_CODE,
  'http://hl7.org/fhir/ValueSet/observation-interpretation' as
  OBS_INTERPRETATION_SYST
from
  lab_result_cm labs
  left join PCORNET_FHIR_MAPPING tcc1 on tcc1.column_cd='RESULT_MODIFIER'
  and labs.RESULT_MODIFIER=tcc1.local_in_cd
  left join PCORNET_FHIR_MAPPING tcc2 on tcc2.column_cd='ABN_IND' and
  labs.ABN_IND=tcc2.local_in_cd

In contrast with the i2b2 mapping exercise, we found that we had more gaps and mismatches to handle between PCORnet and FHIR owing to PCORnet’s much stricter data model. Mapping results fell into 3 categories: (1) variable and/or value set was mappable and was mapped; (2) variable and/or value set was mappable and will be mapped in a future CAMP FHIR release; or (3) variable and/or value set has no equivalent (or no exact equivalent) in FHIR and cannot be mapped either partially or fully. A list of variables and value sets in the third category of PCORnet data are provided in Tables 3 and 4.

**Figure 1:**
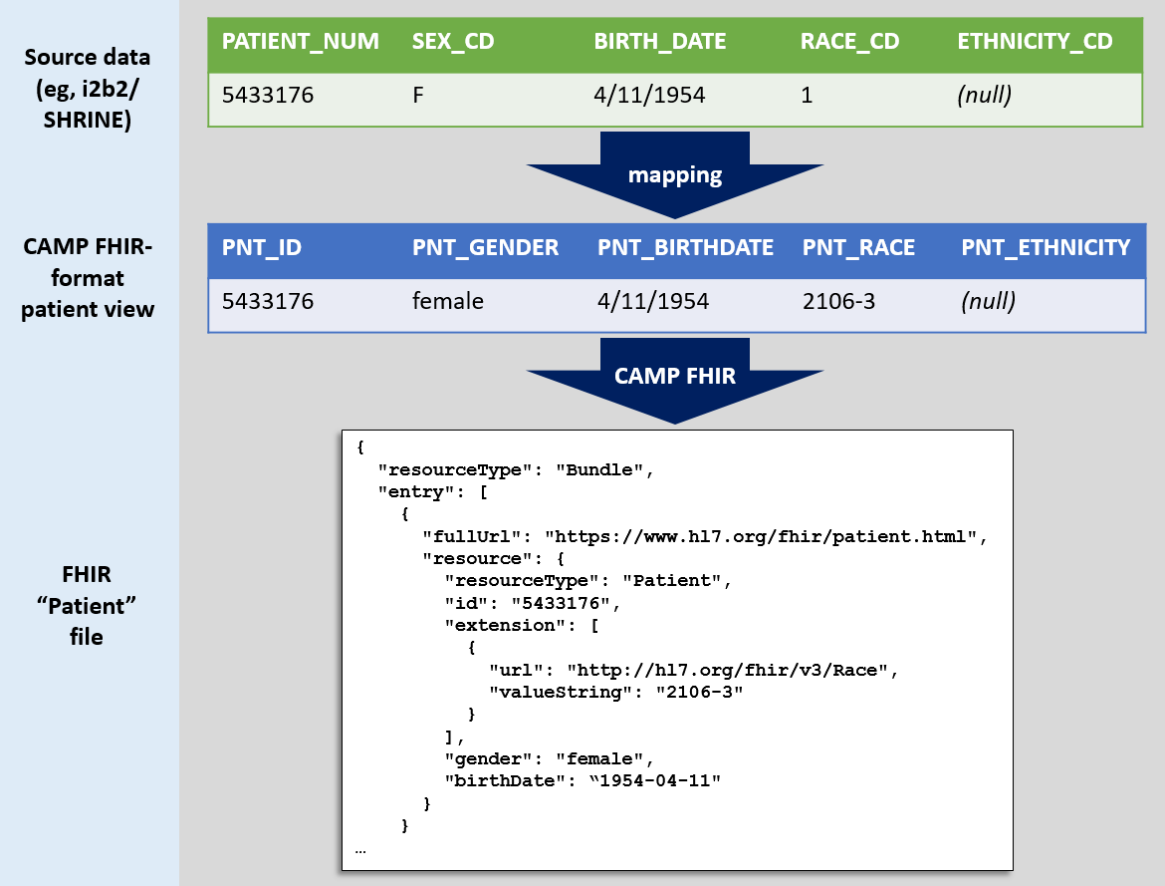
An example of demographic data transformation. CAMP FHIR: Clinical Asset Mapping Program for Fast Healthcare Interoperability Resources; i2b2: Informatics for Integrating Biology & the Bedside.

Regardless of source data model, we operationalize the Hibernate mappings using the open-source HAPI-FHIR API, which is an implementation of the HL7 FHIR specification for Java. HAPI-FHIR supports all versions of FHIR, although CAMP-FHIR currently supports FHIR version 3. Taken together, this setup allows CAMP FHIR to read in the mapped data (via Hibernate), convert to the FHIR standard (via HAPI-FHIR), and output valid FHIR files in XML or JavaScript Object Notation (JSON) format. This process is illustrated with fictitious data in Figure 1.

CAMP FHIR is intended to transform CDM data for a given cohort, rather than an entire warehouse of EHR data. We have found performance to be quite efficient with a predefined cohort, as detailed in Table 5.

**Table 3:** Patient-Centered Outcomes Research Network 4.1 data with no (noncustom) exact Fast Healthcare Interoperability Resources equivalent.

Table^a,b^	Field(s) with no Fast Healthcare Interoperability Resources (FHIR) equivalent
DEMOGRAPHIC	SEXUAL_ORIENTATION, GENDER_IDENTITY, BIOBANK_FLAG
DIAGNOSIS	DX_ORIGIN, DX_POA
PROCEDURE	PX_SOURCE
VITAL	VITAL_SOURCE, BP_POSITION, TOBACCO^c^, TOBACCO_TYPE
LAB_RESULT_CM	RESULT_LOC
PRO_CM	Entire table cannot be mapped
PRESCRIBING	RX_SOURCE
DEATH	Entire table (other than DEATH_DATE) cannot be mapped
DEATH_CAUSE	Entire table cannot be mapped

^a^This table is inclusive of all PCORnet 4.1 fields that did not map to one of the FHIR resources accounted for in the *current version* of CAMP FHIR, which does not include all PCORnet fields. There may be additional unmappable fields uncovered in future versions of CAMP FHIR. Current resources are: Patient, Encounter, Condition, Procedure, Observation, MedicationRequest, and Practitioner.

^b^PCORnet 4.1 tables not intended to hold EHR data are not accounted for here: ENROLLMENT, PCORNET_TRIAL, and HARVEST

^c^Note that this refers specifically to smokeless tobacco. Smoking status is mappable.

**Table 4:** Patient-Centered Outcomes Research Network 4.1 value sets with no (noncustom) exact Fast Healthcare Interoperability Resources equivalents.

Value set^a^	Comment
DEMOGRAPHIC.RACE	No Fast Healthcare Interoperability Resources (FHIR) value for multiple races
ENCOUNTER.ENC_TYPE	No FHIR equivalent for visits of type EI (emergency department admit to inpatient hospital stay), IC (institutional professional consult)
ENCOUNTER.DISCHARGE_STATUS	Imperfect FHIR equivalents for several discharge statuses; 17 possible values in Patient-Centered Outcomes Research Network (PCORnet) versus 11 in FHIR; values were mapped where possible.
ENCOUNTER.ADMITTING_SOURCE	Imperfect FHIR equivalents for several admitting sources; 16 possible values in PCORnet versus 10 in FHIR; values were mapped where possible.

^a^PCORnet 4.1 values of *No information*, *Unknown*, and *Other* were rarely mappable to FHIR and are not noted each time.

**Table 5:** Clinical Asset Mapping Program Fast Healthcare Interoperability Resources’s (CAMP FHIR) performance extracting data from the Patient-Centered Outcomes Research Network common data model.

Domain	Time to populate database view^a^ (seconds)	Time to write JavaScript Object Notation files to disk (seconds)	Number of records
Patient	6	6	15,945
Condition	480	415	2,766,556
Encounter	200	115	1,010,823
Observation (Labs)	390	350	2,081,826
Observation (Vitals)	360	250	1,663,897
Medication Request	450	420	2,435,813
Practitioner	7	7	36,749
Procedure	80	80	442,921

^a^Database server specifications: OS: Red Hat Enterprise Linux Server release 6.10 (Santiago), Processor: Intel(R) Xeon(R) CPU E5-2690 v2 @ 3.00GHz, Database: Oracle 12.1.0.2.0 (Enterprise Edition), Database memory_target: 2 GB, Database size: 464 GB.

## Results

### Asthma Use Case

The JSON-formatted FHIR files output by CAMP FHIR would rarely be the end deliverable for any project. Rather, the FHIR files are a launching point for further transformation, as can be seen in the context of our work with the Translator program. For Translator, we have used CAMP FHIR to extract and transform data from our institution’s i2b2 database on approximately 23,000 patients with asthma, including their associated encounters, laboratory results, vital signs, diagnoses, procedures, medications, and smoking status. (Although this particular use case is using an asthma cohort, the same processes and level of effort would apply to any defined cohort; nothing in the transformation effort described here is specific to asthma.)

**Figure 2:**
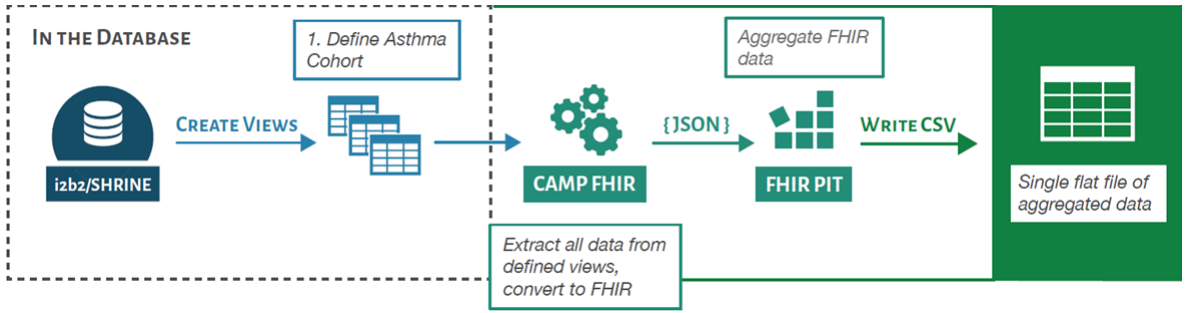
The Clinical Asset Mapping Program fast healthcare interoperability resources (CAMP FHIR) pipeline as used for translator. CSV: comma-separated value; JSON: JavaScript Object Notation; PIT: Patient data Integration Tool.

For this use case, the JSON-formatted FHIR files output by CAMP FHIR were then ingested by a second application, termed FHIR PIT (FHIR Patient data Integration Tool) [23]. FHIR PIT is a custom, open-source application that was developed as part of the Translator program to integrate FHIR-formatted clinical data with environmental exposures data (ie, airborne pollutant exposures, roadway exposures, and socioeconomic exposures) for downstream application in translational research. The resulting data then are accessible via an API endpoint, termed Integrated Clinical and Environmental Exposures Service (ICEES) [24]. As we use FHIR as a standard, any Translator institution or non-Translator institution is able to use CAMP FHIR to transform their source clinical data and use FHIR PIT to provision integrated data via ICEES, with very little local variation. The CAMP FHIR to FHIR PIT to ICEES process is illustrated in Figure 2.

### Validation

To validate the output from CAMP FHIR, we compared the ICEES clinical data generated by the CAMP FHIR/FHIR PIT pipeline with equivalent clinical data for the same patient cohort extracted directly from UNC Health Care System’s enterprise data warehouse, the Carolina Data Warehouse for Health (CDWH). The validation process included the generation of summary statistics for each variable from the 2 data files, including patient counts, mean values, standard deviations, and quartile values. As we iterated through the validation process, we did encounter issues with the software that needed to be corrected. For example, we initially identified inconsistencies in medication data, which we discovered was because of the fact that our i2b2 instance uses RxNorm to code medications, and our warehouse uses nonstandard medication IDs (generated by our EHR). This issue was resolved by referencing a crosswalk between RxNorm codes and our internal medication IDs to translate between the 2 code sets. Any other similar issues causing inconsistencies between the 2 datasets were ultimately discovered and resolved.

In the end, we were able to successfully demonstrate that the final ICEES output of clinical data from CAMP FHIR/FHIR PIT matched exactly the raw extract of clinical data from the CDWH (ie, our validation test, after code corrections, demonstrated 100% accuracy in the mappings). A total of 53 ICEES fields were mapped to FHIR, including 3 Encounter resource mappings on ED and inpatient visits, 4 patient resource mappings on demographics, 1 Observation resource mapping on BMI, 25 condition resource mappings on diagnoses, and 20 MedicationRequest resource mappings on prescribed medications. Note that the particular set of fields chosen did not include any field that was unmappable or resulted in a loss of granularity, thus ensuring a faithful translation for this particular use case. A list of the ICEES fields is available on the ICEES GitHub repository [25].

## Discussion

### Principal Findings

We have shown that CAMP FHIR is a sound method for conversion of relational clinical data to the HL7 FHIR format. On the basis of our experience with the Translator program and our participation in various clinical data research networks, we believe that for certain projects, the use of CAMP FHIR to harmonize clinical data across institutions will save resources over the alternative of standing up matching CDMs. Moreover, because we have made our mapping work public, we are hopeful that new users of CAMP FHIR will not always need to undertake this mapping effort themselves, so long as they use one of CAMP FHIR’s supported CDMs. If a CAMP FHIR user wanted to build a new set of mappings to support an entirely new data model, 4 weeks of effort split among two informaticians (with appropriate understanding of the CDM in question) would be a reasonable estimate for the amount of effort required to map value sets and variables and perform peer review.

The biggest challenge encountered during the mapping process was limiting subjectivity as much as possible, which we handled with peer review, and resolving mapping decisions where disagreements occurred. Another challenge (with no immediate solution) was determining how to handle valid source system data with no match in FHIR—eg, if a patient’s race is *multiple*, without the exact races specified, FHIR has no standard way of storing that information. That means the patient’s race becomes null in the FHIR version of their record, unless custom values or extensions are used. Depending on the use case or research question, these losses could be significant. At this time, the way to handle this issue is through documentation, so that the user understands what data can and cannot be represented through the CAMP FHIR process.

Indeed, we did find examples of loss of data (where source data have no good equivalent in FHIR), change of data meaning (where FHIR equivalents are close, but not an exact match), or loss of granularity (where FHIR value sets have less detail than source value sets). These issues are not uncommon in data transformation in general, and are certainly not limited to transformations to FHIR. In particular, FHIR’s current lack of coverage for data on cause of death, patient reported outcomes, genomics, or patient gender identity (to pick a few examples) may disqualify it for use in answering certain research questions. It is important, then, to put data mapped to FHIR (or any transformed data) in its proper context, and acknowledge that at present, FHIR is likely not ready (yet) to be a single source of truth for clinical research data. For a given use case, if highly granular detail from the source system is important to the research question and that detail is lost during transformation to FHIR, then CAMP FHIR may not be sufficient in and of itself for that study. In short, no data model is the right choice for all applications. This should particularly be taken into account where institutions have no choice as to which data model to use, such as studies that must use CDISC ODM/SDTM standards for FDA compliance. However, our hope is that FHIR’s breadth of data domains and wide adoption would allow it to serve a large variety of use cases, if not all.

Despite its many potential benefits for data harmonization, output from CAMP FHIR would not serve as a replacement for CDMs (and certainly not enterprise clinical data warehouses). Rather, CAMP FHIR output is better suited to handling data for a defined cohort, particularly in the context of a multi-institutional collaboration involving multiple CDMs. If a participating institution is able to take advantage of the prepackaged mapping scripts included with CAMP FHIR, CAMP FHIR will reduce, though not eliminate, cost and effort barriers to participation in such a collaboration. Although there is no particular size limitation on such a cohort, attempting to store millions of patient records in FHIR files could be unwieldy from a file-size and data-manipulation perspective. However, that limitation alone does not discount FHIR’s value as a potential data persistence model, even if the data to be persisted cover individual patient cohorts. The adoption of FHIR as a persistence model is strengthened by the reality that many organizations can export data directly from their EHR using ubiquitous FHIR APIs, thus obviating any translation pathway through other CDMs. This assumes the institutions can agree upon a consistent version of FHIR, which, as is the case with many CDMs, can cause mismatched schemas even within the same data model. Assuming such version agreement is possible, academic medical centers might leverage such a FHIR persistence layer to consolidate data from legacy CDMs, ongoing EHR updates, and accretions from research protocols.

If an institution is capable of natively outputting FHIR files from its EHR, whereas a collaborator prefers to use a CDM as its data source, there is no reason why the native FHIR output could not be combined with the CAMP FHIR output. This provides additional CAMP FHIR use cases—eg, to support rare data sharing opportunities, such as collaborations between academic medical centers and community hospitals. As EHRs increasingly adopt FHIR as a standard for data transmission, it will be far more likely for nonacademic clinical organizations to be able to produce FHIR-formatted data using their EHR than they are to stand up an instance of i2b2, PCORnet, or OMOP, which are more commonly found at academic medical centers. The ability to combine CAMP FHIR output with native FHIR could thus help to democratize the opportunity to participate in data-driven clinical research.

### Future Work

Future work will look beyond data harmonization toward the variety of ways in which the output from CAMP FHIR can be used. FHIR output is intended to be used in a variety of downstream applications, as was done as part of the Translator program with ICEES. Other possibilities include consumption and display by a Web application, consumption by an EHR, or conversion to another data format such as Resource Description Framework (RDF). RDF is an example of another interoperability-focused technology that may prove useful in interinstitutional clinical data sharing in the near future. In this context, CAMP FHIR would thus be situated as middleware between raw clinical data and its ultimate use case.

On the basis of the successful implementation and application of CAMP FHIR at our institution, another logical next step is to formally evaluate its performance at another institution for further testing and validation. A critical metric to track will be the amount of local configuration (and effort) necessary to run the application at an outside institution, as users should only need to make minimal changes to implement the pipeline locally. In general, the more *strict* the source CDM (eg, PCORnet), the less we expect local variation to necessitate mapping changes. Less strict CDMs may require more local changes, though the structure of the queries and the *FHIR side* of the mappings should remain constant. In the near future, we plan to (1) add additional views in future releases to cover more FHIR resources, such as Coverage, Location, Medication Dispense, and Medication Administration; (2) build in OMOP mappings; and (3) introduce support for FHIR version 4.0.

### Conclusions

The Translator program envisions a future in which the entire range of biomedical data, from clinical data to data derived from chemistry, genomics, anatomy, and beyond, is accessible within a unified framework. Such a framework will allow translational research questions to be formulated and answered via query and computation over federated, interoperable data models. As part of the Translator program, we saw a need for unifying heterogeneous clinical data models from collaborating institutions. CAMP FHIR was motivated by a need to foster the sharing of clinical data across Translator institutions for downstream applications in translational research. As CAMP FHIR’s utility ultimately extends beyond the Translator use case, we anticipate its adoption and use across the CTSA consortium and other clinical and translational research collaborations facing a need to harmonize clinical data.

## Abbreviations

ACT: Accrual to Clinical Trials Network
CAMP FHIR: Clinical Asset Mapping Program for FHIR
CDM: common data model
CTSA: Clinical and Translational Science Award
EHR: electronic health record
FHIR PIT: Fast Healthcare Interoperability Resources Patient data Integration Tool
FTE: full-time equivalent
HL7 FHIR: Health Level 7 Fast Healthcare Interoperability Resources
i2b2: Informatics for Integrating Biology & the Bedside
ICEES: Integrated Clinical and Environmental Exposures Service
JSON: JavaScript Object Notation
LOINC: logical observation identifiers names and codes
NC TraCS: North Carolina Translational and Clinical Sciences Institute
OMOP: Observational Medical Outcomes Partnership
PCORnet: Patient-Centered Outcomes Research Network
RDF: Resource Description Framework
SNOMED CT: Systematized Nomenclature of Medicine—Clinical Terms
STAR: Stakeholders, Technology, and Research Clinical Research Network
UNC: University of North Carolina
